# Early Neurological Deterioration following acute stroke: association with reperfusion therapies and National Institutes Of Health Stroke Scale score

**DOI:** 10.3389/fstro.2025.1518685

**Published:** 2025-01-30

**Authors:** Tony Bing Yu, Cameron Lee, Mohammed Mallah, Caroline Domingos Belo, Maria Lucia Uribe Mz Recaman, Yassine Noui, Samantha Bayhonan, Beatrix Sari, Yee-Haur Mah

**Affiliations:** ^1^King's College Hospital NHS Foundation Trust, London, United Kingdom; ^2^School of Medicine, St George's, University of London, London, United Kingdom; ^3^Department of Medical Sciences, Uppsala University, Uppsala, Sweden; ^4^School of Biomedical Engineering and Imaging Sciences, King's College London, London, United Kingdom

**Keywords:** acute stroke, Endovascular Thrombectomy (EVT), Intravenous Thrombolysis (IVT), Early Neurological Deterioration (END), National Institutes Of Health Stroke Scale (NIHSS) score, reperfusion therapies

## Abstract

Early Neurological Deterioration (END) following acute stroke is associated with worse long-term functional outcomes. END is poorly defined and its relationship to reperfusion therapies is not fully understood. NIHSS is commonly used to risk-stratify and identify END following acute stroke however its relationship to END is relatively unexplored. The electronic health record of 933 stroke patients admitted to the Hyperacute Stroke Unit at King's College Hospital in 2022 were manually reviewed for END up to 14-days post stroke to: (1) characterize etiology and risk factors associated with END following acute stroke, and (2) evaluate the association between END, reperfusion therapy and NIHSS. Age, sex and co-morbidity were not associated with END, whereas reperfusion therapy was associated with greater END risk. Admission NIHSS was associated with END in those receiving conventional therapy alone, however, was not associated with END in those receiving reperfusion therapy. For those receiving IVT or EVT, the change in NIHSS at 24-hours was associated with END whereas admission NIHSS was not. In patients with a stable NIHSS 24-hours post stroke, there remained a greater than 10% risk of END. In conclusion, demographic factors and co-morbidity appear less important in determining END risk than stroke severity and treatment type. Admission NIHSS had limited association with END risk in those undergoing reperfusion therapy whereas the change in NIHSS at 24-hours was useful. NIHSS alone appears insufficient in its sensitivity to END to act as a risk-stratification tool, as significant END risk remains in those with stable or improving NIHSS.

## Introduction

The prevalence of stroke in the UK is ~100,000 cases per year (Stroke Statistics, 2024) and it ranks as the third leading cause of combined morbidity and mortality worldwide (Feigin et al., 2021). Acute ischemic strokes (AIS) account for about 80% of all strokes (O'Donnell et al., 2010) and can be managed with reperfusion therapies: Intravenous Thrombolysis (IVT) or Endovascular Thrombectomy (EVT). Their increased use has revolutionized hyperacute stroke care, significantly improving functional outcomes. Haemorrhagic strokes, which comprise 20% of all strokes, are generally managed conservatively or with neurosurgical intervention in more severe cases.

In cases of acute neurological injury, patients are vulnerable to neurological deterioration within the first few days following the event (Shi et al., 2023). In stroke, Early Neurological Deterioration (END) is associated with several pathological processes, including haemorrhagic transformation, cerebral oedema, haematoma expansion, and thrombus propagation (Cuadrado-Godia, 2015) which can manifest as new focal neurological symptoms and/or a reduced conscious level. Management escalation may involve repeat imaging, transfer to intensive care, or neurosurgical intervention, with many poor 90-day outcomes in AIS after reperfusion therapy associated with END (Shi et al., 2023; Che et al., 2022).

SNOMED CT is a comprehensive, standardized medical terminology developed to improve clinical data sharing. It allocates a unique SNOMED CT code to medical concepts like diagnoses, procedures, and medications, and is widely used in electronic health records (EHR) and health data analytics to support data interoperability and global health reporting.

Unfortunately, END is not defined in the SNOMED CT nor ICD-10 libraries, meaning most patients will not have this label associated with their admission. Whether clinical deterioration is classified as END depends on the underlying cause and severity of the symptoms. Most definitions suggest occurrence within the first 72-h post stroke (Cuadrado-Godia, 2015). Incidence is estimated to be between 5 and 40% (Liu et al., 2023) and is linked to worse functional outcomes (Che et al., 2022). Many studies have therefore investigated factors associated with END risk and its relationship with reperfusion therapies (Girot et al., 2020; Yu et al., 2020), aiming to develop predictive models to accurately identify END before it occurs (Gong et al., 2020; Miyamoto et al., 2017; Xie et al., 2021).

In an effort to standardize and facilitate data curation, several definitions have been proposed using established clinical impairment scores. Frequently END has been defined with a threshold of a 2 or 4 point increase in the National Institute of Health Stroke Scale (NIHSS) score (Seners et al., 2015). There are, however, limitations to using NIHSS score, including poor specificity for certain etiologies of END, inadequate representation of posterior circulation-related symptoms, and low sensitivity.

Also, although the NIHSS score can be used with a temporal resolution of hours (Che et al., 2022), in many UK institutions, it is usually only performed after reperfusion therapy, and at 24 h intervals. Considering the limitations of the NIHSS, the curation of large datasets currently requires manual review of patient notes, which is a labor-intensive and challenging task. Consequently, there are relatively few studies, especially from the UK and Western Europe, addressing this vulnerable population.

In this study, we conduct one of the largest retrospective reviews of electronic notes at a tertiary stroke center with access to IVT and EVT to identify early neurological deterioration in acute stroke patients, and to investigate its relationship to reperfusion therapies by examining medical history, standardized stroke scores, clinical assessments, and underlying etiologies.

## Methods

### Data collection

Adult patients admitted with Acute Stroke to the Hyperacute Stroke Unit at King's College Hospital NHS Foundation Trust in 2022 were retrospectively reviewed. Of the 1,026 admissions identified using the Sentinel Stroke National Audit database, access to the electronic health record (EHR) was available for 933. Each admission was manually reviewed for episodes of deterioration up to 14 days post stroke. Episodes of deterioration underwent repeat review by a Stroke consultant and were qualified as END or non-END. Downgrading episodes highlighted as potential deterioration but not deemed to qualify on adjudication removed any “false positive” episodes. To estimate a “false negative” rate we randomly selected 100 “no deterioration” cases for second review, of which there were no missed episodes.

### Defining END

Episodes of deterioration were recognized through changes in clinical status as recorded in the EHR. We used broad indicators to ensure episodes were captured, including clinical documentation and changes in vitals, GCS, and/or NIHSS. END episodes were defined as worsening of neurological status and radiological evidence when available was used to support or refute END.

### Statistical analysis

Statistical analysis was performed in MATLAB (Inc TM, 2020) and R (R Core Team, 2024). Descriptive statistics included mean with standard deviation (SD) and median with interquartile range (IQR). Rates of END between groups were compared using Fisher Exact test or Mann-Whitney U test. Univariate logistic regression was performed to assess the probability of END based on NIHSS at baseline, NIHSS at 24 h, change in NIHSS, delay to treatment and age, overall and by treatment subgroup.

## Results

**Figure 1:**
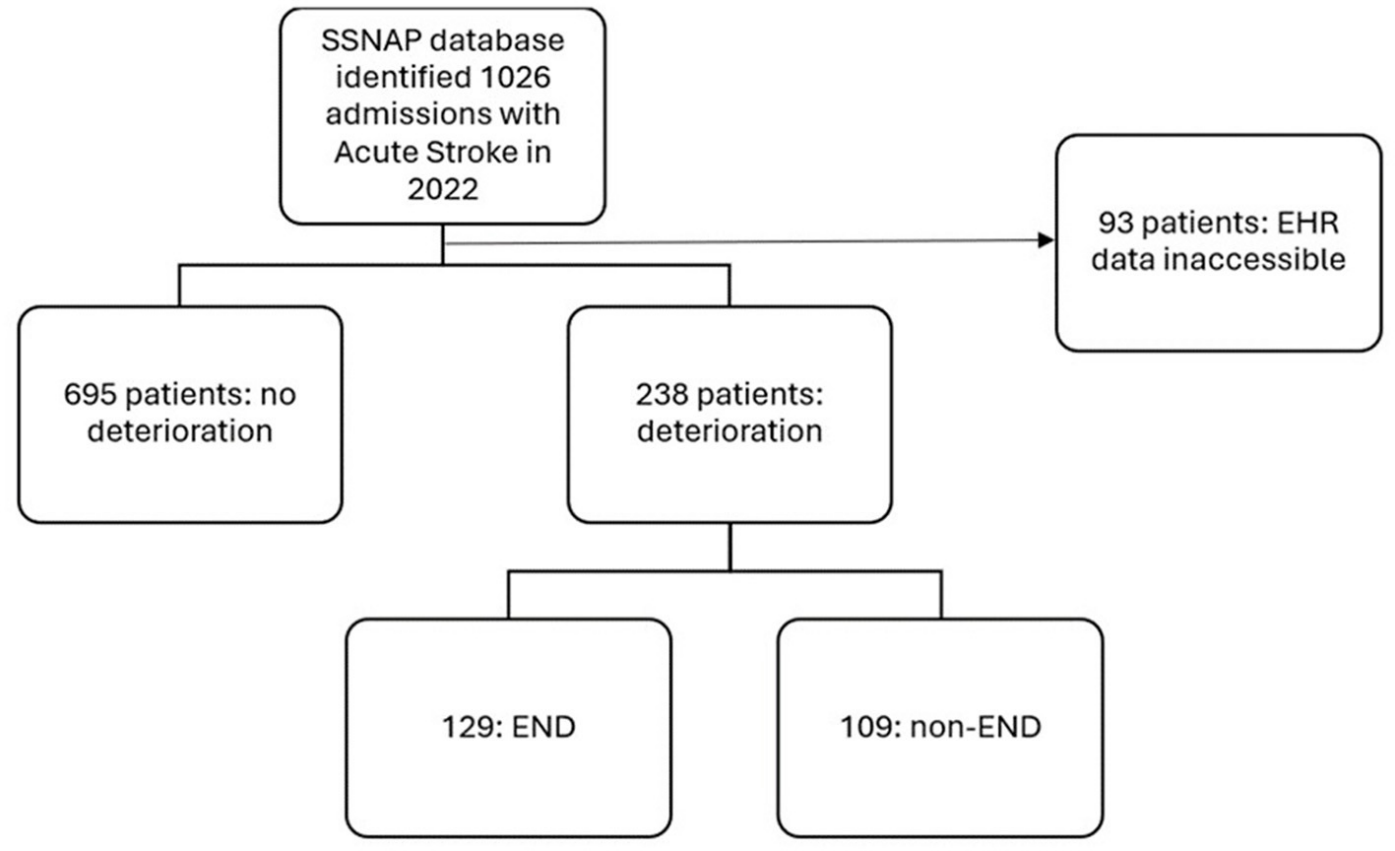
Flowchart outlining patient selection, frequency of post-stroke deterioration and frequency of those qualifying as early neurological deterioration. SSNAP, Sentinel Stroke National Audit Programme; EHR, Electronic Health Records.

Of the 933 patients in this cohort, 129 (13.8%) developed END (Figure 1) and there were 146 total episodes of END amongst these patients. 52.7% of END episodes occurred within the first day, 86.0% occurred within the first 3 days and 98.4% occurred within the first 7 days (Supplementary Figure 1).

Of the 129 patients who developed END, at presentation 38 had carotid/M1, 15 had M2, and 1 had an M3 occlusion. No large vessel occlusion was identified at presentation for 62 patients, of which 31 (50%) presented as hemorrhage. One patient presented as a venous sinus thrombosis with acute thalamic infarction, and the remaining 12 were posterior circulation strokes (Supplementary Table 1).

The most frequent END etiology was cerebral oedema accounting for 21.2% of episodes, followed by seizure (19.2%) and haemorrhagic transformation (13.0%). Together these top three etiologies account for >50% of END in this cohort. Whilst repeat stroke (10.2%), stroke progression (7.5%) and clot propagation (5.5%) each occurred at slightly lower frequency, but cumulatively represent a significant proportion (Supplementary Table 2A).

Occurrence of END was not significantly different based on age, sex, pre-morbid modified ranking scale (mRS), or co-morbidity; the presence of hypertension, congestive cardiac failure, atrial fibrillation, or diabetes, were not associated with END occurrence (Supplementary Table 2A). Overall, arrival NIHSS was significantly greater when comparing patients who developed END to those who did not (Supplementary Table 2B).

**Figure 2:**
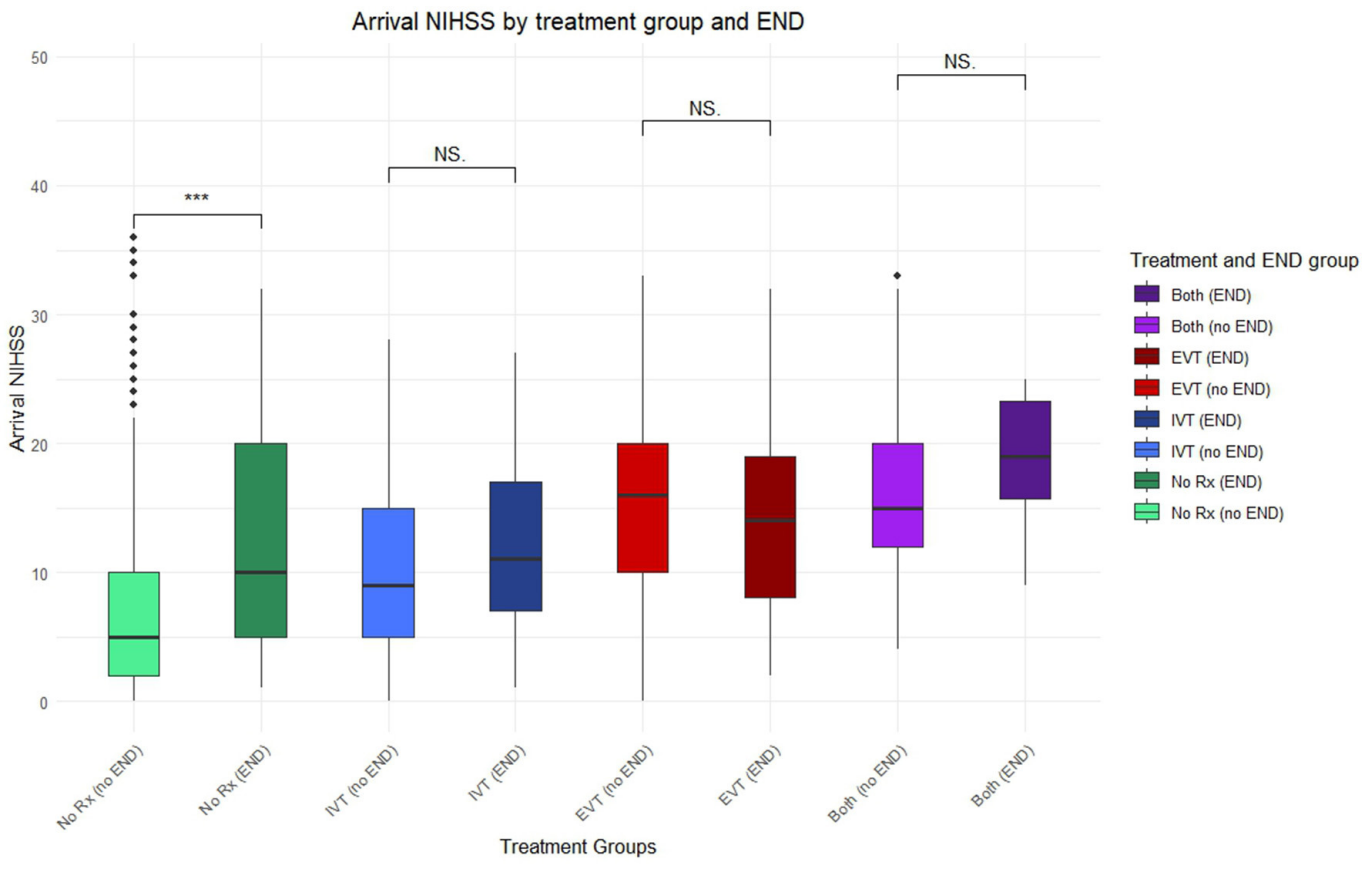
Boxplot of arrival NIHSS paired between different treatment groups and incidence of END. Points lying outside 95% confidence interval marked with additional diamonds. Significant finding *p* < 0.05 (***), NS, not significant; No Rx, no treatment; IVT, intravenous thrombolysis; EVT, endovascular thrombectomy; either thrombolysis or thrombectomy (any), both thrombolysis and thrombectomy (both).

Out of 933 patients, 706 received no reperfusion therapy (75.7%), 137 received IVT alone (14.7%), 89 received EVT alone (9.54%) and 94 patients received both IVT and EVT (10.1%). There was a significantly higher incidence of END in those receiving reperfusion therapy against those who did not. Mean arrival NIHSS was lowest in those receiving no reperfusion therapy, and highest in those receiving either EVT or both IVT and EVT (Figure 2). Arrival NIHSS was not significantly associated with END in any reperfusion therapy group, whereas it was associated with increased END risk in those receiving conventional treatment alone (the “no treatment” group) (Table 1A). The change in NIHSS at 24-hours was associated with greater END risk in those receiving EVT alone (*p* = 0.005) and appeared to approach significance in those receiving IVT alone (*p* = 0.051) (Table 1B).

**Table 1:** END Association with NIHSS by treatment group.

	**OR (95% CI)**	***p*-value**
**(A) Arrival NIHSS**		
All patients	1.06 (1.04–1.08)	< 0.001^*^
No Treatment	1.08 (1.05–1.11)	< 0.001^*^
IVT alone	1.05 (0.97–1.12)	0.18
EVT alone	0.98 (0.92–1.05)	0.61
Both	1.08 (0.96–1.22)	0.19
**(B) Change in NIHSS**		
Any treatment	1.08 (1.04–1.12)	< 0.001^*^
IVT alone	1.08 (1.00–1.17)	0.051
EVT alone	1.09 (1.03–1.17)	0.005^*^
Both treatments	1.08 (0.99–1.18)	0.10

Univariate logistic regression analysis of END based on: arrival NIHSS **(A)** and change in NIHSS over 24-h **(B)**, overall and by treatment group.

No Rx, No treatment; IVT, intravenous thrombolysis; EVT, endovascular thrombectomy; any, either thrombolysis or thrombectomy; both, both thrombolysis and thrombectomy. ^*^Statistically significant.

**Figure 3:**
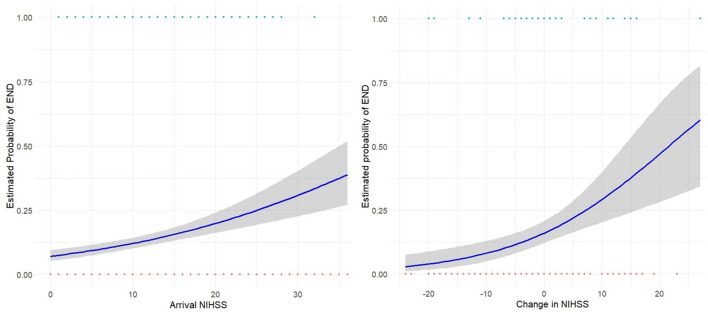
Logistic Regression curves showing estimated probability of END based on arrival NIHSS (**left**) for the entire study cohort, and change in NIHSS at 24-h (**right**) for those receiving reperfusion therapy. Gray shading shows 95% CI.

Overall, the estimated probability of END based on logistic regression analysis increased gradually with both an increase in arrival NIHSS and a greater NIHSS change at 24 h (Figure 3). Interestingly, there remained greater than 10% risk of developing END with a static or even improving NIHSS (Figure 3, right).

## Discussion

END following acute stroke is associated with increased disability and mortality over the short and long-term, making it an important entity to characterize. We retrospectively evaluated END in a large cohort of 933 patients admitted to a single tertiary stroke center with acute stroke, to characterize the timing, etiology and clinical correlates, of END.

The reported incidence of END varies widely. In our cohort, the incidence of END following reperfusion therapy was 13.8%, which aligns closely with that found in a large meta-analysis focusing on post-reperfusion therapy (Shi et al., 2023). Fewer studies have examined END in patients who did not receive reperfusion therapy, with reported incidences ranging from 13.3% to 36.8% (Seners et al., 2015). Our finding of END in 12% of patients who did not receive reperfusion therapy is consistent, albeit at the lower end.

We found no significant association between age, sex, or comorbidities and END, aligning with some previous studies (Rashid et al., 2022; Yang et al., 2024), though others have reported links with age, AF, and hypertension (Shi et al., 2023; Liu et al., 2023; Gong et al., 2020). The lack of association between comorbidity and risk of END observed was surprising given the known stroke risk certain comorbidities confer. For example, the risk of cardioembolic stroke due to AF would be expected to rise in the immediate post stroke period when anticoagulants are held, and in some cases, thrombolysis has been delivered. Indeed, early recurrent ischaemic stroke has been associated with AF, albeit as a rarer END cause (Awadh et al., 2010; Georgiadis et al., 2006).

We used a post-stroke window of 14 days in which episodes of END would be included to capture all potentially relevant episodes. Shorter intervals ranging from 24-72hours (Shi et al., 2023; Girot et al., 2020; Li et al., 2023; Jin et al., 2023) have been used in several studies, while others have extended to 7 days (Liu et al., 2023; Gong et al., 2020; Yang et al., 2024; Xu et al., 2023) or more (Kim et al., 2015). We observed that almost all episodes of END occurred within the first seven days with a majority within three days, indicating that at least a 72-h window may be preferable in studying END. Clinically, 72 h is the typical target duration of care in a hyperacute stroke setting in the UK (NHS National Stroke Programme, 2021) and our findings are consistent with this representing the high-risk period in which closer monitoring is necessary.

Interestingly, in a study of 75 AIS patients receiving both baseline and 3–5 day post-stroke MRI, it was shown that patients with early neurological stability (ENS)—defined as a change in NIHSS between ±3—had significantly higher rates of secondary injury on imaging than those with early neurological recovery (ENR) (defined as an NIHSS improvement of ≥4). Importantly, the ENS group had worse long-term outcomes, indicating that clinically significant neurological deterioration occurred in patients without a worsening of 4 points on the NIHSS, and in some cases, with a static or moderately improved NIHSS score (Irvine et al., 2016).

The complexity of characterizing END stems from its diverse underlying etiologies. We found over 75% of END cases were attributed to cerebral oedema, seizures, haemorrhagic transformation, recurrent stroke, or stroke progression. A systematic review found approximately half of END cases result from hemorrhage, malignant oedema, recurrent stroke, or seizures (Seners et al., 2015). In a multicentre study of nearly 2,000 stroke patients, ~70% of END episodes were due to oedema, hemorrhage, or stroke progression (Weimar et al., 2005). These findings suggest that despite aetiological diversity, a few common causes account for a significant proportion of END cases.

By including AIS patients regardless of treatment, we could compare the interaction between different therapies and END. We observed higher rates of END in patients receiving any reperfusion therapy, particularly those undergoing EVT alone or in combination with IVT. Patients receiving IVT alone did not have a significantly higher rate of END compared to those receiving no reperfusion therapy, suggesting that EVT specifically may increase the risk of END. While EVT complications might contribute to this risk, stroke severity, which influences EVT candidacy, likely also plays a role.

In EVT patients, stroke severity appeared to have less influence on END risk. We found no significant difference in arrival NIHSS between END and non-END groups among those receiving EVT or both EVT and IVT. In contrast, in patients receiving no reperfusion therapy or IVT alone, arrival NIHSS was significantly higher in the END group. We hypothesize that EVT's impact may be substantial enough to overshadow any moderate contribution of arrival NIHSS. Supporting this, we observed that the change in NIHSS at 24-h was associated with END overall, particularly in the EVT subgroup. In contrast, this association was not significant in patients receiving no reperfusion therapy or IVT alone. In EVT patients, the change in NIHSS likely reflects the intervention's impact, with significant increases or decreases in NIHSS correlating with heightened or reduced END risk, respectively.

Also, the underlying status of the brain parenchyma following such an insult is likely to contribute and may be reflected in admission (and post intervention) imaging. Further work comparing similar patients who have and have not undergone EVT will be helpful in further defining this relationship.

The relationship between admission NIHSS and END is inconsistently reported in the literature. Several studies have linked higher arrival NIHSS with increased END risk, and at least two have incorporated it into END risk prediction models (Liu et al., 2023; Gong et al., 2020; Xie et al., 2021; Kim et al., 2015; Haeusler et al., 2011). However, these studies often include AIS patients regardless of reperfusion therapy. A systematic review focusing on reperfusion therapy outcomes found no association between baseline NIHSS and END risk (Shi et al., 2023).

Our findings indicate that while arrival NIHSS is associated with an increased risk of END overall, this is not consistent across reperfusion therapy subgroups. This suggests specific interactions between NIHSS, reperfusion therapy, and END. Alternatively, a ceiling effect might occur in reperfusion subgroups due to the exclusion of patients with very low NIHSS, who could drive the overall association between END and arrival NIHSS.

Interestingly, one meta-analysis of IVT outcomes found the highest rates of END in the lowest arrival NIHSS group (Hou et al., 2019). Although the authors noted this might be influenced by prolonged door-to-needle time in one study, this relationship has been observed elsewhere. A retrospective multicentre study of 566 patients who underwent thrombolysis also found that a lower admission NIHSS was associated with an increased risk of END (Mori et al., 2012).

The relationship between arrival NIHSS and END is complex; inconsistencies may reflect genuine differences between patient subgroups or result from artifacts of the scale. Defining END as an NIHSS change of 2 or 4 points means higher scores require a smaller proportional change to qualify as END. Additionally, given that some NIHSS changes may be due to interrater variability, higher NIHSS scores are more likely to show a 2 or 4-point change within the margin of error. Thus, defining END purely by NIHSS change may bias the diagnosis toward patients with higher initial NIHSS.

We identified a group in whom NIHSS was stable or even improved and yet END still occurred. Our results indicate an estimated probability of END with no change in NIHSS of ~10–15%, demonstrating known limitations in the sensitivity of NIHSS. The NIHSS score's relative blindness to posterior circulation signs is well established (Makharia et al., 2024) and subtle deficits and seizures are potential manifestations of END that may not alter NIHSS. The finding of significant rates of secondary neurological injury in post stroke patients exhibiting early neurological stability (Irvine et al., 2016), are consistent with our observations and have important clinical and research implications. Decisions regarding monitoring and transfer of patients based on stable NIHSS may be falsely reassuring in the subset of patients whose stable or improving NIHSS does not reflect their END risk.

Assessing the relationship between change in NIHSS and END is clearly circular if accepting the paradigm that END *is* change in NIHSS. In the present study we avoided setting an NIHSS threshold in defining END, opting instead to qualify episodes based on expert interpretation of various modalities, which could include, but were not limited to NIHSS. We believe the strength of this approach is that it can account somewhat for the heterogeneity of END, identifying cases with stable NIHSS and discounting cases where NIHSS change is not due to END.

The varied definitions of END pose significant challenges. The absence of a dedicated SNOMED CT or ICD-10 code, make large-scale patient reviews cumbersome. While NIHSS is a useful surrogate for detecting END, it has limitations and should not be solely relied upon. Manual reviews, although comprehensive, are time-consuming and lack standardization, complicating comparisons across studies.

Integrating multiple factors could improve END risk estimation but requires large sample sizes. Stratifying risk based on specific etiologies might be more effective, as ICD-10 coded diagnoses can be efficiently extracted from records, while SNOMED CT codes can be inferred from the electronic medical records with AI tools methods (Shek et al., 2021), and could streamline data collection, allowing for more efficient identification of END patients and enabling analysis of multiple predisposing factors.

Although a few common etiologies account for the majority of END cases, using AI to combine detailed manual approaches with large-scale NIHSS-based studies could improve sensitivity and specificity in detecting END. This approach would also ensure that research on END prediction and prevention does not overlook patients whose risk is not adequately reflected by NIHSS scores.

## Conclusion

We have conducted a large retrospective analysis of END at a major London Stroke Unit. Approximately 10% of our dataset exhibited END and the vast majority occurred within 72-hours of stroke onset. Although there is a variety of etiologies, 50% of all END episodes were attributable to either: cerebral oedema, seizure and haemorrhagic transformation. Arrival NIHSS is limited in assessing risk of END in those undergoing reperfusion therapy and specifically those receiving EVT. The change in NIHSS at 24-h is more associated with END risk in these groups, however NIHSS alone is limited with a significant subset of patients remaining at risk of END with a stable of improving NIHSS score.

Reperfusion therapies, arrival NIHSS scores and co-morbidities appear to provide information regarding the risk of END, but their predictive power is diminished by the mix of etiologies encompassed with current methods of defining END. To improve our understanding of this serious phenomenon, and anticipate its occurrence, larger datasets that include patients who both do and do not deteriorate will be necessary. Restricting the definition of END to its underlying etiology, may offer an avenue to leverage developments in AI to support data curation, and group cases that likely share the same pathophysiology.

## Data Availability

The complete, non-patient sensitive data supporting the conclusions of this article will be made available by the authors upon request, without undue reservation.
