# Supplementary material for: Early Neurological Deterioration following acute stroke: association with reperfusion therapies and National Institutes Of Health Stroke Scale score
Source: Front Stroke. 2025 Jan 30;4:1518685. doi: 10.3389/fstro.2025.1518685 (PMC12802648; doi:10.3389/fstro.2025.1518685)
Supplement: Supplementary file 1 [file Table_1.docx]

Supplemental Material


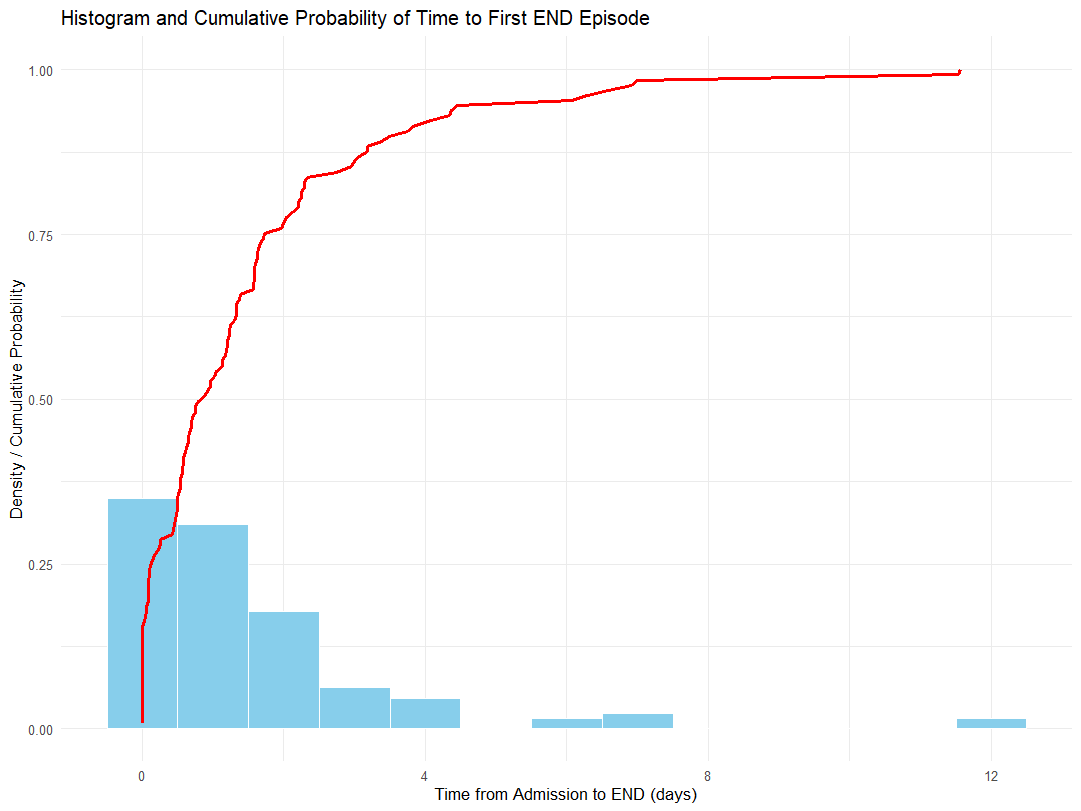


**Supplemental Figure 1:**

Histogram (bars) and Cumulative probability (line) showing duration from admission to first episode of END for the cohort in days.

**Supplementary Table 1: Vessel status in END patients**

| Level of occlusion at presentation | Frequency |
| --- | --- |
| No large vessel occlusion | 31 |
| Carotid/M1 | 38 |
| M2 | 15 |
| M3 | 1 |
| Posterior circulation occlusion | 12 |
| Venous sinus thrombosis | 1 |
| Haemorrhage | 31 |
| Total | 129 |

Table showing the frequency of vessel occlusion categorised by location when present, or alternative aetiology when not present, in patients who exhibited END

**Supplemental Table 2: END aetiologies and demographics**

**A**

| END Cause | Frequency (%) |
| --- | --- |
| Cerebral Oedema | 31 (21.2) |
| Seizure | 28 (19.2) |
| Haemorrhagic Transformation | 19 (13.0) |
| Repeat Stroke | 15 (10.3) |
| Haematoma Expansion | 14 (9.59) |
| Stroke Progression | 11 (7.53) |
| Aspiration pneumonia | 10 (6.85) |
| Clot progression | 8 (5.48) |
| Infection | 4 (2.74) |
| Hydrocephalus | 2 (1.37) |
| Hypoperfusion | 1 (0.68) |
| Other | 3 (2.05) |

**B**

| No END vs END | p-value |
| --- | --- |
| Age | 0.88 |
| Arrival NIHSS | <0.001* |
| Premorbid mRS | 0.09 |
| Sex | 0.26 |
| Congestive Cardiac Failure (CCF) | 0.69 |
| Hypertension | 0.38 |
| Atrial Fibrillation | 1.0 |
| Diabetes | 0.09 |
| Stroke type (AIS vs ICH) | 0.03 |

(A) Table of END aetiologies by subtype, frequency and percentage.

(B) Table comparing demographics and co-morbidities between patients who did and did not exhibit END. P-values were calculated using the Fisher-Exact test.

Abbreviations: Acute Ischaemic Stroke (AIS), Intracerebral haemorrhage (ICH)
